# α‐tocopherol pretreatment alleviates cerebral ischemia‐reperfusion injury in rats

**DOI:** 10.1111/cns.13814

**Published:** 2022-03-18

**Authors:** Shitao Lv, Haiyan Yang, Pengcheng Jing, Haiying Song

**Affiliations:** ^1^ Department of Emergency Yantaishan Hospital Yantai China; ^2^ Yantai City Detoxification Center Yantai China; ^3^ Department of Gynecology Yantai Yuhuangding Hospital Yantai China

**Keywords:** apoptosis, cerebral, inflammation, ischemia‐reperfusion injury, oxidative stress, α‐tocopherol

## Abstract

α‐tocopherol showed antioxidant, anti‐inflammatory and anti‐apoptotic abilities in rat brain tissue, thus alleviating cerebral ischemia‐reperfusion injury‐induced nerve damage.

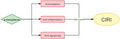

## CONFLICT OF INTEREST

The authors declared no conflict of interest.

Dear Editors,

Stroke is an acute cerebral circulatory disorder of the central nervous system.^1^ It is recognized as the third leading cause of death in the world.^2^ Stroke includes ischemic and hemorrhagic cerebrovascular diseases, with high morbidity, disability, and mortality.^3^ Ischemic cerebrovascular disease (ICVD) accounts for 60%–80% of the stroke and is the main type of cerebrovascular disease.^4^ The incidence of ICVD is increasing year by year, and the mortality rate is as high as 60%–80%.^5^ Even if the patient survives, there are serious sequelae that seriously affects the life quality.^5^ Ischemic brain injury includes primary injury during ischemia and secondary injury during reperfusion.^6^ When the hypoperfused brain tissue gets blood supply again, the ischemic injury does not alleviate and further aggravates, and even more serious consequences such as nerve cell death and fatal cerebral edema appear.^7^ This phenomenon is called cerebral ischemia‐reperfusion injury (CIRI).^7^ CIRI can be secondary to various surgical traumas during the perioperative period, such as cardiopulmonary cerebral resuscitation, neurosurgery, cardiopulmonary bypass, and intraoperative hypotension caused by insufficient cerebral perfusion, which may lead to long‐term learning, memory, and cognitive dysfunction.^8^ For primary ischemic injury, thrombolytic treatment can be given in the therapeutic time window clinically to timely restore blood supply,^9^ but for secondary CIRI, the current clinical treatment and curative effect are far from expected.^9^ Therefore, the pathogenesis and prevention measures of perioperative CIRI have become a scientific problem to be solved urgently. It is currently believed that CIRI is not the result of a single influencing factor, but a complex chain reaction of multiple factors, involving oxidative stress, inflammation, mitochondrial damage, intracellular calcium overload, and blood–brain barrier destruction.^10^ These pathogenesis mechanisms act on CIRI individually or in parallel.^10^ A recent study found that inflammation triggered by high mobility group protein 1–induced CIRI through the nuclear factor kappa‐B (NF‐κB) pathway.^11^ In addition, the regulation of oxidative stress on neuroinflammation and vascular response after CIRI has also been verified.^12^ Vitamin E (VE) has always been considered a nutrient closely related to normal reproduction, so it is also called tocopherol.^13^ α‐tocopherol (α‐TOH) is the highest and most active form of VE in the human body.^14^ It plays an important role in the improvement of the respiratory system, the construction of cognitive ability, and the development of the nervous system.^15^ α‐TOH has shown excellent anti‐inflammatory and antioxidant effects on many diseases.^16^, ^17^ α‐TOH was found to protect heart function through anti‐inflammatory and antioxidation in myocardial ischemia‐reperfusion injury.^16^ In addition, α‐TOH was found in a clinical study to reduce the level of oxidative stress in children with Down syndrome.^17^ However, the inhibitory effect of α‐TOH on inflammation and oxidative stress was rarely studied in CIRI. Therefore, we treated rats with α‐TOH after CIRI and studied its effect on rat CIRI. α‐TOH showed a good alleviation effect on the brain damage of rats after CIRI.

This study was approved by the Animal Ethics Committee of Yantaishan Hospital Animal Center. Eighty male Sprague‐Dawley rats (8–9 weeks old, 220–250 g) were provided by Yantaishan Hospital Experimental Animal Center. The rats are kept in an SPF animal room with a room temperature of 22°C –24°C and a relative humidity of 50%–60%. The artificial 24‐h day–night cycle of light and good ventilation was maintained in the animal room. Rats can eat and drink freely. Rats were randomly divided into the Sham+normal saline (NS) group, Sham+α‐TOH group, CIRI+NS group, and CIRI+α‐TOH group. Rats in the Sham+NS group and Sham+α‐TOH group were made surgical incisions without modeling. Rats in the CIRI+NS group and CIRI+α‐TOH group were used to construct rat models of global cerebral ischemia and reperfusion by the modified Pulsinelli four‐vessel occlusion method. Rats in Sham+α‐TOH group and CIRI+α‐TOH group were injected with α‐TOH (100 mg/kg) (Selleck) subcutaneously daily in the week before operation.^18^ Rats in the Sham+NS group and CIRI+NS group were injected with the same amount of NS daily in the week before operation.

After 7 days of drug treatment, a modified Pulsinelli four‐vessel occlusion method was used to construct a rat brain CIRI model.^19^ The rats were fasted 8 h before the operation. The rats were anesthetized with 2% sodium pentobarbital (40 mg/kg) and fixed on the operating table. The rat was maintained anesthesia by intermittent administration of sevoflurane, and the breathing of the rat was maintained through the tracheal intubation. The rats were injected with sodium lactate Ringer's injection (1 ml/h) through the tail vein to keep systemic circulation. After disinfecting the rat's neck skin, we excised the skin in the front and middle area of the rat's neck and isolated bilateral common carotid arteries. We used 20G silicone tube to insert from mouse nasal cavity and inject 0.9% sodium chloride solution. After the hippocampus temperature dropped to 33°C ± 0.5°C, the bilateral common carotid arteries of the rats were ligated for 15 min. During the whole process, electroencephalogram, electrocardiogram, and the temperature of hippocampus were recorded to monitor the rats. The rats were killed by cervical dislocation 24 h after surgery. Neurological deficit score (NDS) was evaluated by the Spinal Cord Assessment Tool published by Akpinar et al.^20^ A score of 0 refers to good nerve function; a score of 1 refers to the disorder of the flexion and extension of the rat's forelimbs when the rat's tail is lifted; a score of 2 refers to the failure of the rat to maintain balance when walking, but it can be at rest; the score of 3 refers to the failure of the rat to maintain balance when stand up; and a score of 4 means that the rat cannot walk spontaneously and completely loses consciousness. The rat brain tissue was preserved at −20°C for 30 min. The brain tissue was then made into coronal sections, each with 2‐mm thickness. The sections were placed in a 2% TTC solution (Sigma‐Aldrich) and incubated at 37°C for 20 min. The sections were then fixed with 4% paraformaldehyde for 24 h. The pale part was considered as the area of the infarction. The mass of fresh brain tissue was wet weight. The brain tissue was then placed in a drying oven at 110°C. When the deviation of the mass of three consecutive measurements was within 0.2 mg, the brain tissue was considered to be completely dry and the mass at this time was dry weight. Water content = (wet weight − dry weight)/wet weight × 100%. γ‐aminobutyric acid was determined by liquid chromatography–mass spectrometry. Liquid chromatography analysis was ACQUITY‐Ultra Performance Liquid Chromatography system (Waters MS Technologies). The mass spectrometer was Micromass Q‐TOF (Waters MS Technologies). The determination of γ‐aminobutyric acid was carried out by the Qingxi Technology Research Institute. An appropriate amount of brain tissue was lysed and centrifuged in a centrifuge (12,000 rpm, 5 min, 4°C). The supernatant was collected, and the adenosine triphosphate (ATP) in the supernatant was detected using the ATP assay kit (Abcam) according to the manufacturer's instructions. An appropriate amount of rat brain tissue was collected and homogenized. The activity or content of superoxide dismutase (SOD), glutathione peroxidase (GSH‐Px), nitric oxide synthase (NOS), and nitric oxide (NO) was tested by specific SOD, GSH‐Px, NOS, and NO kits (ThermoFisher) according to the manufacturer's instructions. All samples were heated in a 60°C water bath for 30 min. Hundred microliters of the reaction mixture (ThermoFisher) was added to the standard wells and sample wells. After 5 min, we add 10 μl of nicotinamide adenine dinucleotide (NADH) developer to each well and mix them. After 2 h of incubation, we used microplate reader (Molecular Devices) to detect the absorbance at 450‐nm wavelength. Enzyme‐linked immunosorbent assay (ELISA) was used to detect inflammatory factors in rat serum. We collected an appropriate amount of blood from the rat heart and left it at room temperature for 30 min. After the blood has coagulated, we put it in a centrifuge to extract the serum (12,000 rpm, 5 min, 4°C). Interleukin (IL)‐1β, IL‐6, and tumor necrosis factor (TNF)‐α ELISA kits (ThermoFisher) were used to detect the levels of these inflammatory factors in the serum according to the manufacturer's instructions. An appropriate amount of brain tissue was used to extract total RNA by the TRIzol (Invitrogen) method. RNA was stored at −80°C for long‐term storage. mRNA was reversed to complementary deoxyribose nucleic acid (cDNA) by SuperScript IV Reverse Transcriptase (ThermoFisher). SYBR Green Master Mix (Vazyme) was used to amplify the corresponding cDNA according to different primers. The number of cycle amplification was 40. GAPDH was used as an internal reference. 2^−ΔΔCT^ was used to indicate the relative expression of mRNA. The brain tissue of the rat was made into paraffin sections. After washing the deparaffinized sections with phosphate‐buffered saline (PBS), we added a mixture of TDT and DIG‐D‐UTP (Sigma‐Aldrich) to the sections and left them at 4°C for 2 h. The sections were then blocked for 30 min and incubated with antibodies for 1 h. Finally, we used antifluorescence quenching mounting tablets for mounting and recording the experimental results through a fluorescence microscope. All experimental results are represented as mean ± standard deviation. Statistical Product and Service Solutions (SPSS) 21.0 statistical software (IBM) and Graphpad Prism 7.0 were used to analyze the experimental results of this study. Shapiro–Wilk normality test was used to assess data distribution. If data exhibit a Gaussian distribution, comparison between multiple groups was done using one‐way ANOVA test followed by post hoc test (least significant difference). If not, nonparametric test (Mann–Whitney test) was used. *p* < 0.05 was considered statistically significant. The sample size of all experiments was more than 3.

**FIGURE 1:**
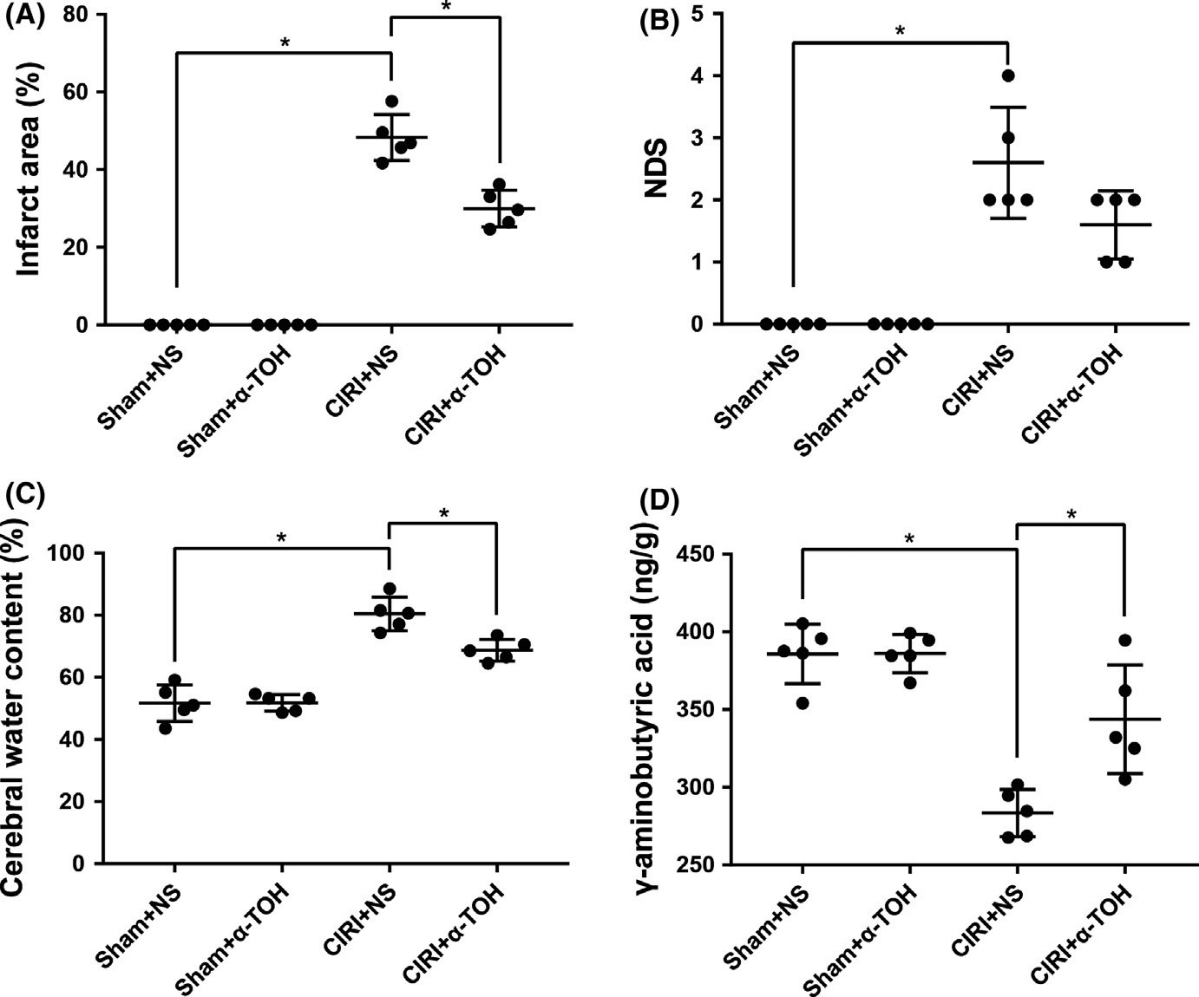
α‐TOH improved nerve damage symptoms in CIRI rats. (A) Infarct area of brain tissue in rats; (B) NDS of rats in four groups; (C) cerebral water content; (D) γ‐aminobutyric acid in brain tissue of rats (“*” means *p* < 0.05)

In order to clarify the effect of α‐TOH on rat nerve damage, we constructed a rat CIRI model by blocking and recanalizing bilateral carotid arteries. TTC staining revealed the severity of cerebral infarction in rats. The rats in the Sham+NS group and Sham+α‐TOH group had no obvious cerebral infarction, while the cerebral infarction area of the rats after modeling was as high as about 48% and 29%. The cerebral infarction area of α‐TOH pretreated rats was significantly lower than that of untreated rats (Figure 1A). NDS showed that α‐TOH can partially alleviate the nerve damage in rats (Figure 1B). In addition, the cerebral water content of CIRI rats was significantly higher than that of normal rats, indicating that CIRI rats have obvious brain edema, and α‐TOH pretreatment can significantly reduce the water content (Figure 1C). α‐TOH also made the content of γ‐aminobutyric acid partly rebound (Figure 1D). These results indicated that α‐TOH pretreatment can partially ameliorate the nerve damage induced by CIRI.

**FIGURE 2:**
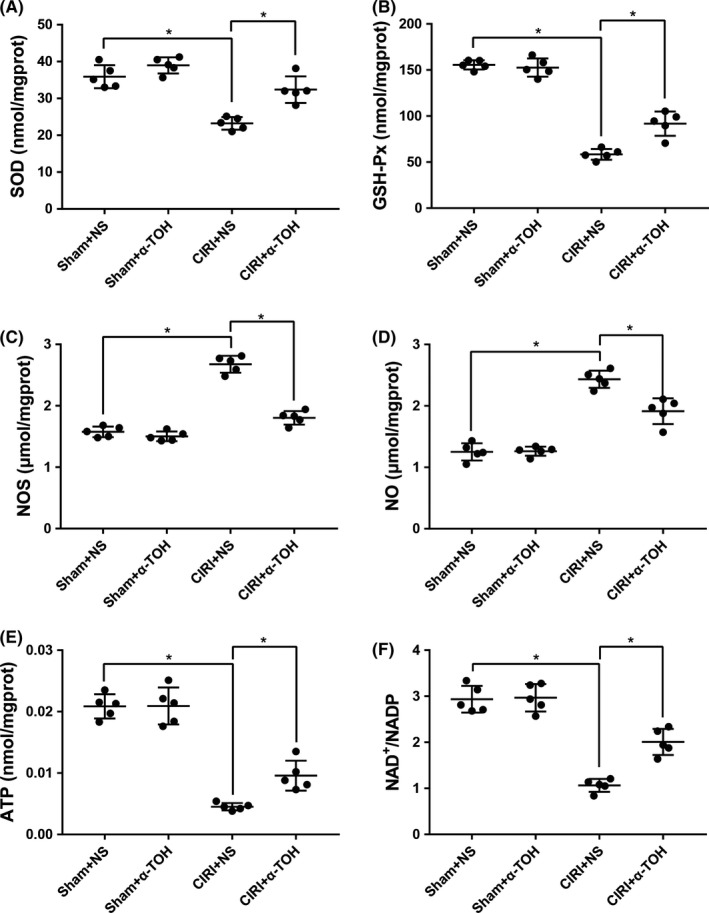
α‐TOH reduced the level of oxidative stress in the brain tissue of CIRI rats. Activities of SOD (A), GSH‐Px (B), NOS (C), NO (D), ATP (E), and NAD^+^/NADP ratio (F) were detected to determine the oxidative stress in the brain tissue of CIRI rats (“*” means *p* < 0.05)

Considering that oxidative damage is one of the main causes of CIRI, we detected changes in the level of oxidative stress in rat brain tissue. After the CIRI model was established, the levels of SOD, GSH‐Px, and ATP in the brain tissue of rats decreased, while the levels of NOS and NO and the ratio of NAD+/NADH increased (Figure 2A–F). The α‐TOH pretreatment partly suppressed these changes. These indicated that α‐TOH pretreatment can partially inhibit CIRI‐induced oxidative stress.

**FIGURE 3:**
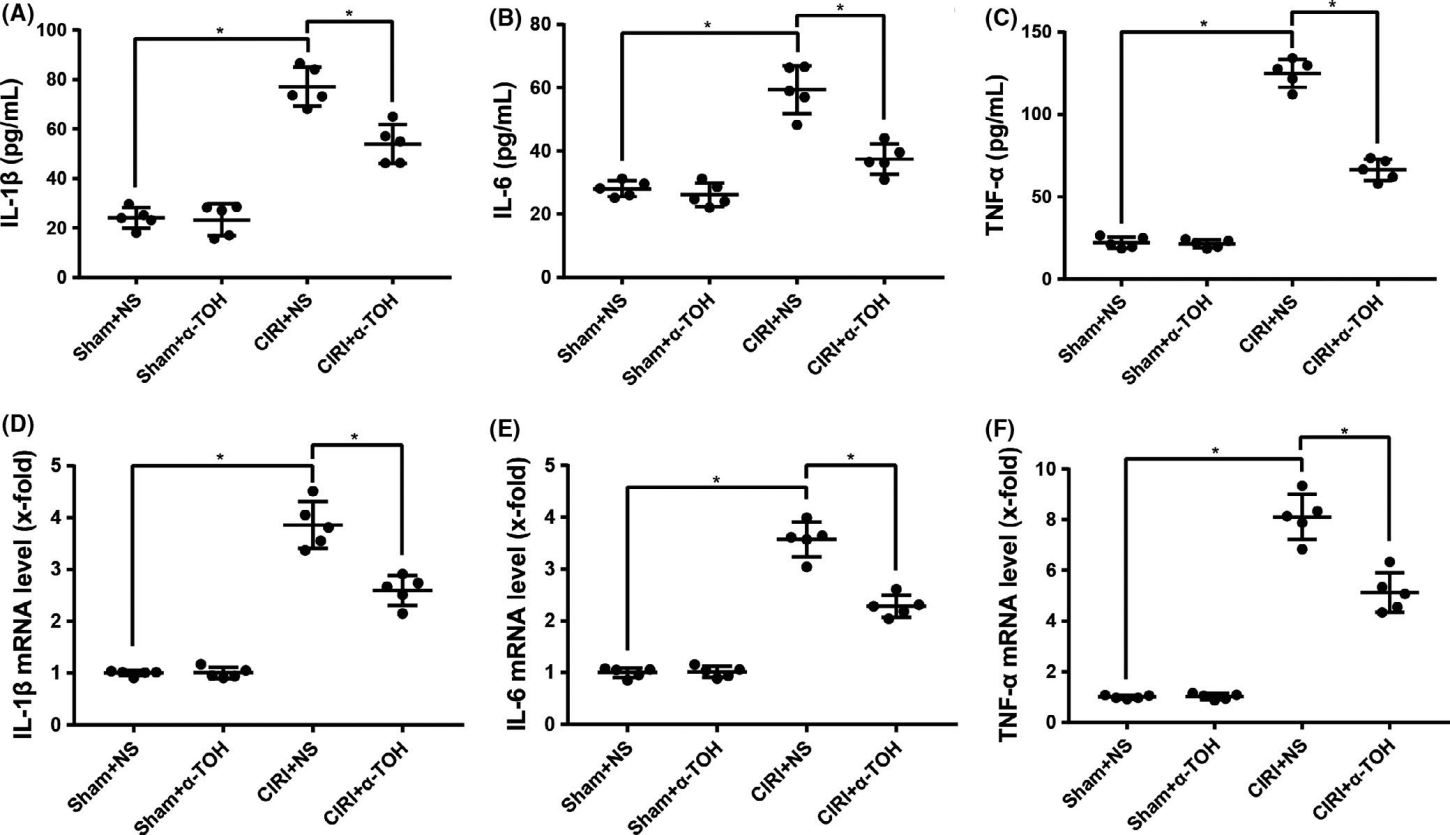
α‐TOH reduced inflammation levels in CIRI rats. IL‐1β (A), IL‐6 (B), and TNF‐α (C) in rat serum were detected by ELISA; mRNA of IL‐1β (D), IL‐6 (E), and TNF‐α (F) in rat brain tissue were detected by RT‐PCR (“*” means *p* < 0.05)

We also detected changes in the level of inflammation in rats. The results of ELISA showed that the inflammatory factors including IL‐1β (Figure 3A), IL‐6 (Figure 3B), and TNF‐α (Figure 3C) in the serum of rats after CIRI increased greatly, and α‐TOH pretreatment effectively reduced these inflammatory factors. In addition, we detected the mRNA of these inflammatory factors in rat brain tissue and found that α‐TOH also reduced the mRNA expression of IL‐1β (Figure 3D), IL‐6 (Figure 3E), and TNF‐α (Figure 3F).

**FIGURE 4:**
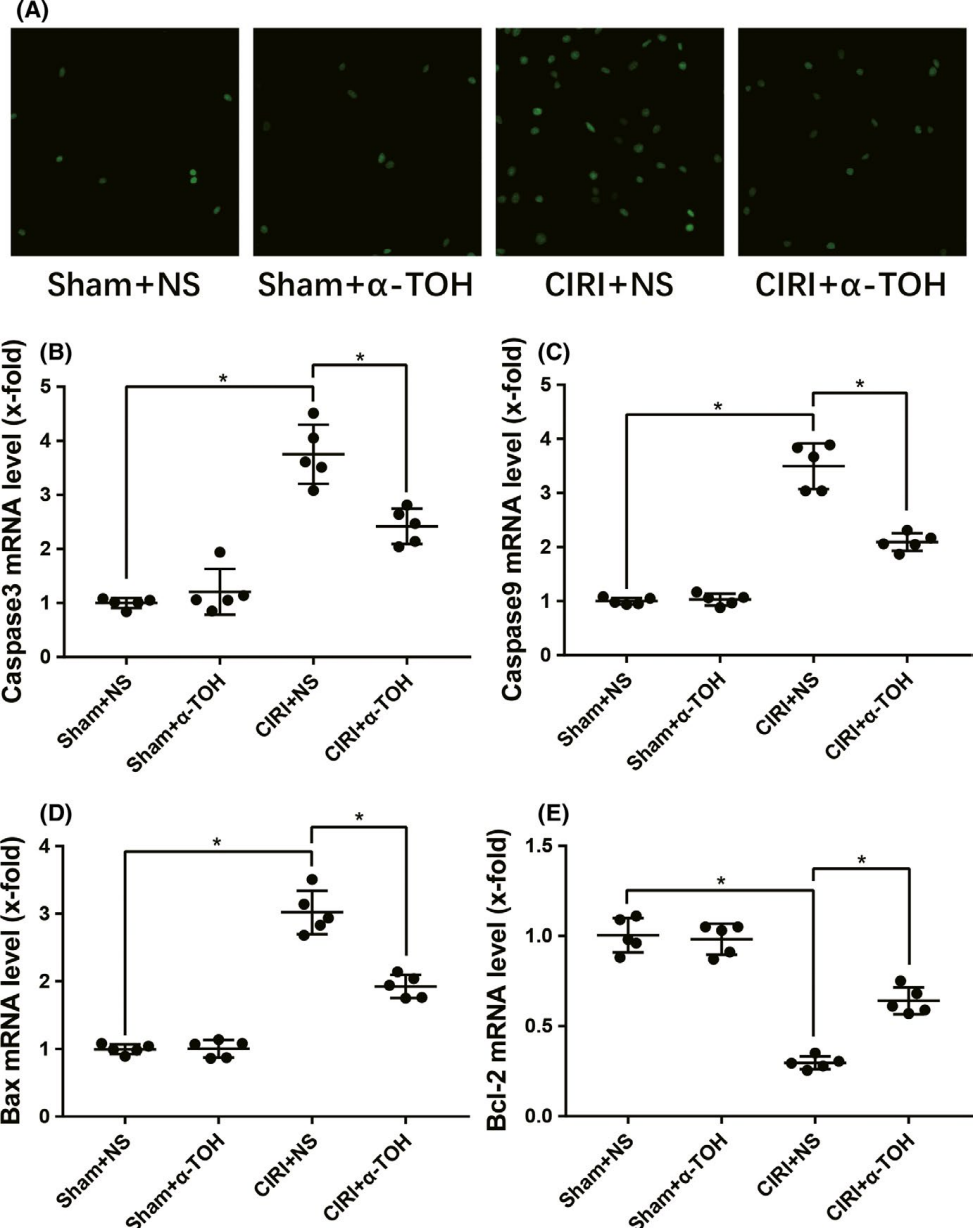
α‐TOH inhibited cell apoptosis in the brain tissue of CIRI rats. (A) TUNEL staining results of brain tissue in rats (magnification: 200×); (B–E) mRNA expression of caspase3, caspase9, Bax, and Bcl‐2 in brain tissue (“*” means *p* < 0.05)

Neuronal apoptosis is an irreversible phenomenon of brain damage, so we detected cell apoptosis in rat brain tissue. TdT‐mediated dUTP nick end labeling (TUNEL) staining detected the apoptosis in the brain tissue of rats, and we found that the level of apoptosis in the brain tissue of rats after CIRI was significantly increased. In the brain tissues of CIRI rats pretreated with α‐TOH, the level of apoptosis was partially reduced (Figure 4A). We also tested the mRNA levels of caspase3 (Figure 4B), caspase9 (Figure 4C), Bax (Figure 4D), and Bcl‐2 (Figure 4E). α‐TOH inhibited the expression of caspase3, caspase9, and Bax and promoted the expression of Bcl‐2 mRNA. This indicated that α‐TOH inhibited the caspase family and reduced the Bax/Bcl‐2 ratio.

The occurrence and development of CIRI is a complicated process.^21^ Oxygen‐free radicals and inflammatory factors are important factors that cause secondary damage.^22^ Oxygen‐free radicals can cause damage to neuron cell membrane structure, protein degeneration and breakage, and then aggravate inflammation damage and cause neuron apoptosis and death.^22^ α‐TOH is a kind of vitamin E, which has excellent antioxidant and anti‐inflammatory effects.^14^ α‐TOH has the characteristics of easy oxidation and can react with oxygen‐free radicals.^15^ Some studies have found that α‐TOH has no toxic side effects at the cellular level and in animal experiments.^23^, ^24^ Our results revealed that α‐TOH reduced inflammatory and oxidative damage in CIRI model rats and therefore reduced neuronal apoptosis. Therefore, α‐TOH may have a protective effect on brain tissues suffering from reperfusion injury.

Under normal physiological conditions, the production and elimination of the body's aerobic metabolite reactive oxygen species (ROS) are strictly controlled and maintained in a balanced state without harm to the body.^25^ When certain reasons (such as pathophysiological changes caused by CIRI) cause this balance to be disrupted, excessive ROS production or failure of the body's antioxidant system, ROS in the body will trigger a series of reactions and damage the body.^26^ The redox reaction caused by the production and release of a large amount of ROS in the CIRI process is a key step in causing nerve cell necrosis and apoptosis.^27^ Therefore, removing a large amount of ROS is important to alleviate CIRI. In theory, any treatment or drug that affects the pathway of ROS production may alleviate CIRI caused by oxidative stress.^28^ In recent years, a large number of studies using CIRI rat models have confirmed the role of antioxidant therapy in the treatment of CIRI, and certain progress has been made.^29^, ^30^ Lee et al.^29^ revealed the important role of maintaining the activity of the antioxidant protein thioredoxin 2 in the treatment of cerebral ischemia. In addition, Kamada et al.^30^ also found that hyperglycemia aggravated the blood–brain barrier disorder after CIRI by decreasing SOD activity. We found that α‐TOH has a significant promoting effect on antioxidants in brain tissue and reduces the production of oxidation products such as NO and NOS, which indicated that the antioxidant capacity of α‐TOH reduces the oxidation of brain tissue in CIRI rats.

Inflammation plays an important role in CIRI. The activation and exudation of inflammatory cells (such as microglia, astrocytes, and white blood cells, especially neutrophils) are key steps in the inflammatory response of the central nervous system.^31^ In stroke and CIRI, the inflammatory response is triggered in the early stage of ischemia, starting from the energy depletion caused by adenosine triphosphate in the early stage of ischemia, and then, oxidative damage and inflammatory response in the reperfusion stage are mutually causal and co‐acting.^31^ Inflammatory factors, inflammatory cells, and inflammatory mediators are involved in the “waterfall” cascade.^31^ The production of inflammatory factors in the brain tissue and serum of rats treated with α‐TOH was significantly reduced. The decrease in the level of inflammation indicated the alleviation of the inflammatory injury in rats after α‐TOH treatment. Similar results have also been found in ischemia‐reperfusion injury in other tissues. Wallert et al.^16^ found that α‐TOH can alleviate cardiac dysfunction caused by myocardial ischemia and reperfusion by inhibiting inflammation. In addition, α‐TOH has also been found to inhibit the activation of macrophages to reduce inflammation.^32^

Apoptosis is an autonomous cell suicide phenomenon initiated by the expression of apoptotic necrosis factors under the regulation of genes.^33^ It is an important way for the metabolism of multicellular organisms and the elimination of abnormal cells. A study has shown that cell apoptosis is closely related to delayed neuronal damage after CIRI and is one of the pathophysiological bases for the further aggravation of nervous system dysfunction after CIRI.^34^ In terms of the molecular genetics, Bcl‐2 protein family, cysteine aspartic protease (caspase) family, NF‐κB, and mTOR are the most important regulatory mechanisms of neuronal apoptosis. Bcl‐2 exerts its antiapoptotic effect by inhibiting the effect of Bax. The expression of Bcl‐2 in CIRI is closely related to neuron survival and has a protective effect on neurons. Bcl‐2 and Bax protein coexist in the cell and are in dynamic equilibrium. The amount of expression of Bcl‐2 and Bax activity determines the outcome of cells under multifactorial injury. Bcl‐2 can form a heterodimer Bax‐Bcl‐2 with Bax to inhibit Bax‐mediated mitochondrial permeability transition (PT) pore and Bax channel opening, so the apoptotic precursor substances cannot enter the cytoplasm to initiate the apoptotic pathway. Therefore, whether the cell tends to survive or die after the apoptosis stimulus signal can be shown by the Bcl‐2/Bax ratio.^35^ Therefore, regulating Bcl‐2/Bax gene expression has important guiding significance for CIRI therapy. In this study, α‐TOH inhibited the expression of Bax and caspase3 family and promoted the expression of Bcl‐2. This indicated that Bcl‐2/Bax is more than 1 under the action of α‐TOH. Therefore, the anti‐inflammatory and antioxidant effects of α‐TOH effectively alleviated cell apoptosis in brain tissue.

In conclusion, this is the first to study the effect of α‐TOH in the CIRI rat model. We first find that α‐TOH may have a good application prospect in CIRI. We believe that our results provide new targets and directions for clinical CIRI treatment.

## Data Availability

The data that support the findings of this study are available from the corresponding author upon reasonable request.
